# (–)-Loliolide Isolated from *Sargassum horneri* Suppressed Oxidative Stress and Inflammation by Activating Nrf2/HO-1 Signaling in IFN-γ/TNF-α-Stimulated HaCaT Keratinocytes

**DOI:** 10.3390/antiox10060856

**Published:** 2021-05-27

**Authors:** Eui-Jeong Han, Ilekuttige Priyan Shanura Fernando, Hyun-Soo Kim, Dae-Sung Lee, Areum Kim, Jun-Geon Je, Min-Jeong Seo, Young-Heun Jee, You-Jin Jeon, Seo-Young Kim, Ginnae Ahn

**Affiliations:** 1 Research Center for Healthcare and Biomedical Engineering, Chonnam National University, Yeosu 59626, Korea; iosu5772@jnu.ac.kr; 2 Department of Food Technology and Nutrition, Chonnam National University, Yeosu 59626, Korea; 3 Department of Marine Bio-Food Sciences, Chonnam National University, Yeosu 59626, Korea; shanurabru@jnu.ac.kr; 4 National Marine Biodiversity Institute of Korea, 75, Jangsan-ro 101 gil, Janghang-eup, Seocheon 33662, Korea; gustn783@mabik.re.kr (H.-S.K.); daesung@mabik.re.kr (D.-S.L.); 5 Department of Veterinary Medicine and Veterinary Medical Research Institute, Jeju National University, Jeju 63243, Korea; orange5687@jejunu.ac.kr (A.K.); yhjee@jejunu.ac.kr (Y.-H.J.); 6 Department of Marine Life Science, Jeju National University, Jeju 63243, Korea; wpwnsrjs@jejunu.ac.kr (J.-G.J.); youjin@jejunu.ac.kr (Y.-J.J.); 7 Freshwater Biosources Utilization Bureau, Bioresources Industrialization Support Division, Nakdonggang National Institute of Biological Resources (NNIBR), Sangju 37242, Korea; minruby@nnibr.re.kr; 8 Interdisciplinary Graduate Program in Advanced Convergence Technology & Science, Jeju National University, Jeju 63243, Korea; 9 Chuncheon Center, Korea Basic Science Institute, Chuncheon 24341, Korea

**Keywords:** (–)-loliolide, antioxidant effects, anti-inflammatory effects, Nrf2/HO-1 signaling, HaCaT keratinocytes

## Abstract

The present study evaluated the effects of (–)-loliolide isolated from *Sargassum horneri* (*S. horneri*) against oxidative stress and inflammation, and its biological mechanism in interferon (IFN)-γ/tumor necrosis factor (TNF)-α-stimulated HaCaT keratinocytes. The results showed that (–)-loliolide improved the cell viability by reducing the production of intracellular reactive oxygen species (ROS) in IFN-γ/TNF-α-stimulated HaCaT keratinocytes. In addition, (–)-loliolide effectively decreased the expression of inflammatory cytokines (interleukin (IL)-4 IL-6, IL-13, IFN-γ and TNF-α) and chemokines (CCL11 (Eotaxin), macrophage-derived chemokine (MDC), regulated on activation, normal T cell expressed and secreted (RANTES), and thymus and activation-regulated chemokine (TARC)), by downregulating the expression of epidermal-derived initial cytokines (IL-25, IL-33 and thymic stromal lymphopoietin (TSLP)). Furthermore, (–)-loliolide suppressed the activation of mitogen-activated protein kinase (MAPK) and nuclear factor-κB (NF-κB) signaling, whereas it activated nuclear factor erythroid 2-related factor 2 (Nrf2)/heme oxygenase-1 (HO-1) signaling. Interestingly, the cytoprotective effects of (–)-loliolide against IFN-γ/TNF-α stimulation were significantly blocked upon inhibition of HO-1. Taken together, these results suggest that (–)-loliolide effectively suppressed the oxidative stress and inflammation by activating the Nrf2/HO-1 signaling in IFN-γ/TNF-α-stimulated HaCaT keratinocytes.

## 1. Introduction

Recently, the incidence of various inflammatory diseases has increased due to many factors, such as diet, stress, and environmental factors [1]. Among human organs, the skin is most closely affected by inflammatory diseases, as it acts as the first line of defense against irritants [2]. In particular, keratinocytes that are mainly present in the external layer of the skin play an indispensable role in protecting against environmental threats [3]. Normally, keratinocytes mediate inflammatory responses by regulating the release of various physiological activators such as cytokines and chemokines [4]. Recent studies have reported that epidermal initial stimulating factors (interleukin (IL)-25, IL-33, and thymic stromal lymphopoietin (TSLP)) are the first to be involved in inflammatory reactions in keratinocytes [5]. In addition, these cytokines activate other inflammatory cytokines (IL-4, IL-6, IL-13, TNF-α and IFN-γ) and chemokines (CCL11 (Eotaxin), macrophage-derived chemokine (MDC), regulated on activation normal T cell expressed and secreted (RANTES), thymus and activation-regulated chemokine (TARC)), thereby leading to more severe inflammation [6].

Oxidative stress is closely related to inflammatory response [7]. Enhanced production of reactive oxygen species (ROS) promotes the migration of inflammatory cells through vasodilation, and causes skin tissue injury [8]. Nuclear factor erythroid 2-related factor 2 (Nrf2)/heme oxygenase 1 (HO-1) signaling, which activates the expression of antioxidant genes, is involved in protecting against oxidation and inflammatory reactions by inhibiting ROS production [9].

Steroids are representative anti-inflammatory drugs, which are used for the treatment of inflammatory skin disorders [10]. However, continuous use of steroids is known to cause side effects, such as diabetes, osteoporosis, and cataracts [11,12]. Therefore, it is necessary to develop safer and more effective therapeutics.

Marine-derived materials are structurally diverse, and have been reported to possess various biological activities, such as anti-diabetic, anti-inflammatory, and antioxidant activity [13]. *Sargassum horneri* (*S. horneri*), the brown algae, has been registered as edible according to the Ministry of Food and Drug Safety (MFDS), and its various physiological activities have been extensively studied [14]; (–)-loliolide derived from *S. horneri* has been extensively studied for its beneficial effects. Furthermore, (–)-loliolide has been found to exhibit antioxidant [15], anti-inflammatory [16], anti-apoptotic [15], and anti-melanogenic activities [17] against fine dust. Although several studies have reported that (–)-loliolide has antioxidant and anti-inflammation effects, the underlying mechanisms are still not well known.

In this study, we evaluated the effects of (–)-loliolide isolated from *Sargassum horneri* (*S. horneri*) against oxidative stress and inflammation, and its biological mechanism in IFN-γ/tumor necrosis factor (TNF)-α-stimulated HaCaT keratinocytes.

## 2. Materials and Methods

### 2.1. Materials

Dulbecco’s modified Eagle medium (DMEM), fetal bovine serum (FBS), penicillin-streptomycin (10,000 U/mL each), and the NE-PER^®^ Nuclear and Cytoplasmic Extraction Kit were purchased from Thermo Fisher Scientific (Waltham, MA, USA). Reagents including 3-(4,5-dimethylthiazol-2-yl)-2,5-diphenyltetrazolium bromide (MTT), zinc protoporphyrin IX (ZnPP), and 2′7′-dichlorodihydrofluorescein diacetate (DCFH2-DA) were purchased from Sigma-Aldrich (St. Louis, MO, USA). Enzyme-linked immunosorbent assay (ELISA) kits for human IL-4, IL-6, IL-13, IFN-γ, and TNF-α were obtained from BioLegend, (San Diego, CA, USA). Other reagents or organic solvents used were HPLC grade with purity >99%. The (–)-loliolide compound used in our experiments was the same one used by Thilina et al., 2019 [16] and Dias et al., 2020 [18].

### 2.2. Measurement of Cell Viability

HaCaT, human epidermal keratinocytes, were cultured in DMEM containing 10% heat-inactivated FBS (56 °C) and 1% penicillin and streptomycin solution. To check the effect of (–)-loliolide on cell viability, cells were seeded (1 × 10^4^ cells/well) in 96-well plates and incubated for stabilization. Cells were then treated with different concentrations of (–)-loliolide and after 1 h, cells were stimulated with IFN-γ/TNF-α. After 24 h, the cells were incubated with 15 μL of MTT stock solution (5 mg/mL) for 4 h to stain the live cells. Formazan crystals in stained cells were dissolved in 150 μL of dimethyl sulfoxide (DMSO) and absorbance was measured at 570 nm using an ELISA reader (Sunrise, Tecan Co. Ltd., Grödig, Austria).

### 2.3. Measurement of Intracellular ROS Production

Intracellular ROS production was measured using the DCFH-DA assay. Briefly, cells (1.6 × 10^4^ cells/well) were seeded in a 96-well plate and incubated for 24 h. After incubation, cells were treated with various doses of (–)-loliolide and stimulated with IFN-γ/TNF-α after 1 h. The generated intracellular ROS were detected using DCFH-DA (0.5 mg/mL in 100% ethanol) at an excitation and emission spectra of 485 nm and 520 nm, respectively, using an ELISA reader [18].

### 2.4. RNA Extraction and RT-PCR

Total RNA was isolated using the RNA elution kit. Total RNA (1 μg) was reverse-transcribed in cDNA using the qScript cDNA synthesis kit. The following PCR conditions were used: 5 min denaturing at 94 °C, 1 min annealing at 55–60 °C and a 20 min extension cycle at 72 °C in a TaKaRa PCR Thermal Cycler (TaKaRa Bio Inc., Otsu, Japan). The primer sequences were adopted from the following publication: [19]. The expression of target genes was normalized against that of GAPDH (internal control; Cell Signaling Technology, Beverly, MA, USA) [20].

### 2.5. Measurement of Inflammatory Cytokine Production

The cells were incubated for 1 h with different concentrations of (–)-loliolide (15.6–62.5 μg/mL) and stimulated with IFN-γ/TNF-α for 24 h. The levels of IL-4, IL-6, IL-13, TNF-α, and IFN-γ in the supernatant were measured using the human ELISA kits as per the manufacturer’s instructions [19].

### 2.6. Western Blot Analysis

Proteins for western blot were isolated from whole cells using the NE-PER^®^ Nuclear and Cytoplasmic Extraction Kit. After equalizing protein concentrations, 40 μg of proteins were electrophoresed by SDS-PAGE on a 12% gel. Resolved protein bands were transferred onto polyvinylidene difluoride membranes (Millipore, Bedford, MA, USA) and blocked with 5% bovine non-fat milk (BD Difco Skim milk powder, Franklin Lakes, NJ, USA) for 90 min. The membranes were probed separately with primary antibodies (1:1000 dilution; nuclear Factor-kappa B (NF-κB), mitogen-activated protein kinase (MAPK), Nrf2/HO-1, and NAD(P)H quinone dehydrogenase 1 (NQO-1; Cell Signaling Technology Inc.) overnight at 4 °C. After incubation, the membranes were incubated with the respective horseradish peroxidase (HRP)-conjugated secondary antibodies (Cell Signaling Technology, Beverly, MA, USA) for 90 min. After washing excess secondary antibodies with Tween 20/Tris-buffered saline, the protein bands were visualized using the enhanced chemiluminescent substrate (ECL, Cyanagen Srl, Bologna, Italy). The levels of each protein were normalized to the levels of β-actin or lamin B (mouse monoclonal antibodies), used as internal controls (Cell Signaling Technology).

### 2.7. Influence of (–)-Loliolide in HO-1-Inhibited Cells

To investigate whether HO-1 activation was influenced by the anti-inflammatory effect of (–)-loliolide in human keratinocytes, MTT and DCFH-DA assays were performed. HaCaT keratinocytes were pretreated with 5 μM zinc protoporphyrin (ZnPP; a HO-1 inhibitor) for 1 h with or without (–)-loliolide and with IFN-γ/TNF-α stimulation. MTT and DCFH-DA assays were performed as described in the above sections.

### 2.8. Statistical Analysis

All results are expressed as the mean ± SE of three independent determinations. Differences between the mean values of each group were assessed by one-way analysis of variance, followed by Duncan’s test using predictive analytics software (PASW) version 21.0 (SPSS, Chicago, IL, USA). Results with a *p* value < 0.05 were considered significant.

## 3. Results

### 3.1. (–)-Loliolide Effectively Incresed the Cell Viability by Suppressing the Intracellular ROS Production in HaCaT Keratinocytes

**Figure 1:**
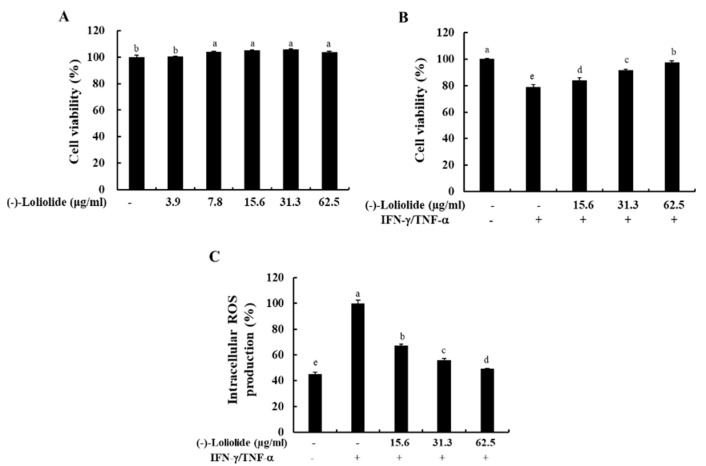
Effects of (–)-loliolide on cell viability (**A**,**B**) and intracellular ROS production (**C**) in IFN-γ/TNF-α-stimulated HaCaT keratinocytes. The results represent data from three independent experiments (n = 3), and the values are indicated as the mean ± SE. Error bars with different letters are significantly different (*p* < 0.05).

To determine the optimal concentration of (–)-loliolide, we performed the MTT assay. As shown in Figure 1A, (–)-loliolide did not induce cytotoxicity at any of the examined concentrations (3.9–62.5 µg/mL). In addition, (–)-loliolide markedly increased the cell viability decreased by IFN-γ/TNF-α stimulation in a dose-dependent manner, and it also effectively inhibited the production of intracellular ROS (Figure 1B,C). These results suggest that (–)-loliolide improved cell viability by decreasing the intracellular ROS production in IFN-γ/TNF-α-stimulated HaCaT keratinocytes.

### 3.2. (–)-Loliolide Downregulated the mRNA Expression Levels of Cytokines and Chemokines in IFN-γ/TNF-α-Stimulated HaCaT Keratinocytes

**Figure 2:**
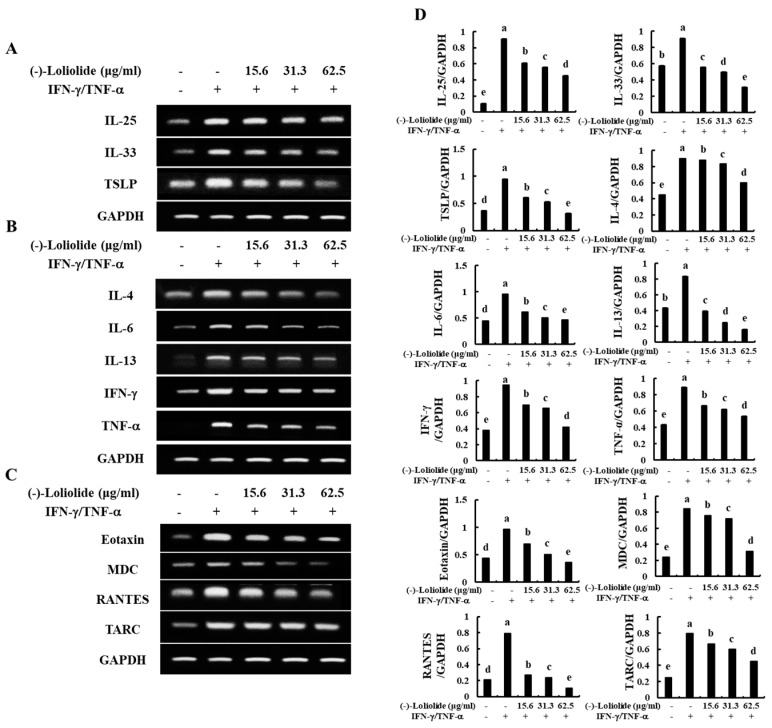
Inhibitory effects of (–)-loliolide on IFN-γ/TNF-α-induced mRNA expression of epidermal stimulating factors (**A**), inflammatory cytokines (**B**), and chemokines (**C**) in HaCaT keratinocytes. Densitometry analysis (**D**) were performed for the three independent experiments (n = 3). Error bars with different letters are significantly different (*p* < 0.05).

As shown in Figure 2A, IFN-γ/TNF-α stimulation increased the mRNA expression levels of epidermal stimulating factors, including IL-25, IL-33, and TSLP. However, (–)-loliolide effectively decreased the expression of these molecules. In addition, (–)-loliolide markedly down-regulated the expression of inflammatory cytokines (IL-4, IL-6, IL-13, IFN-γ, and TNF-α) and chemokines (Eotaxin, MDC, RANTES, and TARC) in IFN-γ/TNF-α-stimulated HaCaT keratinocytes (Figure 2B,C).

### 3.3. (–)-Loliolide Inhibited the Production of Inflammatory Cytokines in IFN-γ/TNF-α-Stimulated HaCaT Keratinocytes

**Figure 3:**
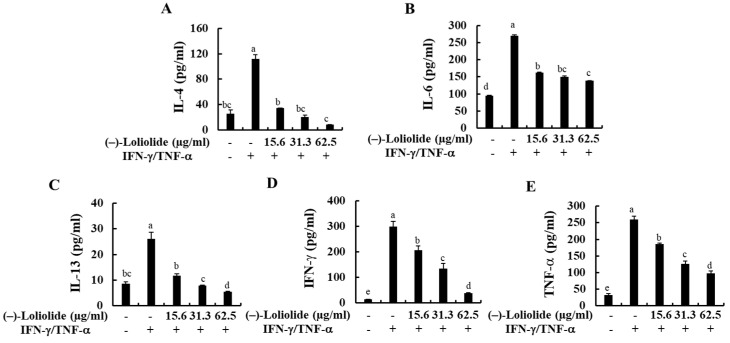
Inhibitory effects of (–)-loliolide on IFN-γ/TNF-α-induced production of inflammatory cytokines (**A**–**E**) in HaCaT keratinocytes. The results represent data from three independent experiments (n = 3), and the values are indicated as the mean ± SE. Error bars with different letters are significantly different (*p* < 0.05).

The production of inflammatory cytokines (IL-4, IL-6, IL-13, IFN-γ, and TNF-α) in IFN-γ/TNF-α-stimulated HaCaT keratinocytes were measured by human ELISA kit. As indicated in Figure 3, IFN-γ/TNF-α stimulation significantly increased the production of inflammatory cytokines, such as IL-4, IL-6, IL-13, IFN-γ, and TNF-α compared to non-treated control cells, whereas they were markedly decreased by the pretreatment of (–)-loliolide. These results suggest that (–)-loliolide effectively suppressed the inflammation caused by IFN-γ/TNF-α stimulation by decreasing the production of pro-inflammatory cytokines.

### 3.4. (–)-Loliolide Inhibited the Activation of MAPK and NF-κB Signaling in IFN-γ/TNF-α-Stimulated HaCaT Keratinocytes

**Figure 4:**
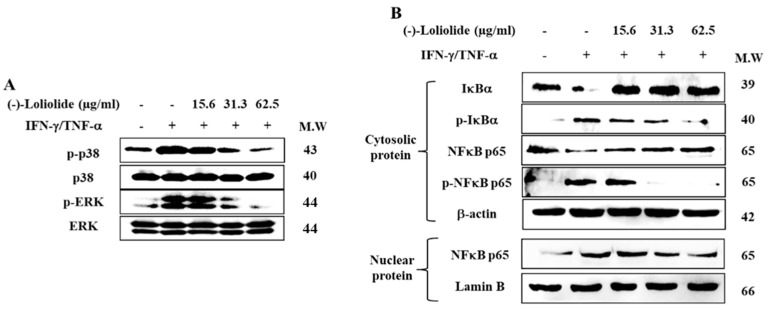
Inhibitory effects of (–)-loliolide on IFN-γ/TNF-α-induced activation of MAPK (**A**) and NF-κB (**B**) signaling in HaCaT keratinocytes.

Western blot was performed to assess the inhibitory effects of (–)-loliolide against the activation of MAPK and NF-κB signaling. The results showed that IFN-γ/TNF-α stimulation resulted in the increased phosphorylation of p38 and ERK, whereas (–)-loliolide effectively decreased them (Figure 4A). Interestingly, (–)-loliolide effectively downregulated the expression of p-IκBα and p65 in the cytosol as well as the translocation of p65 from the cytosol into the nucleus in IFN-γ/TNF-α-stimulated HaCaT keratinocytes (Figure 4B).

### 3.5. Inhibition of HO-1 Reduced the Cytoprotective Effects of (–)-Loliolide in IFN-γ/TNF-α-Stimulated HaCaT Keratinocytes

**Figure 5:**
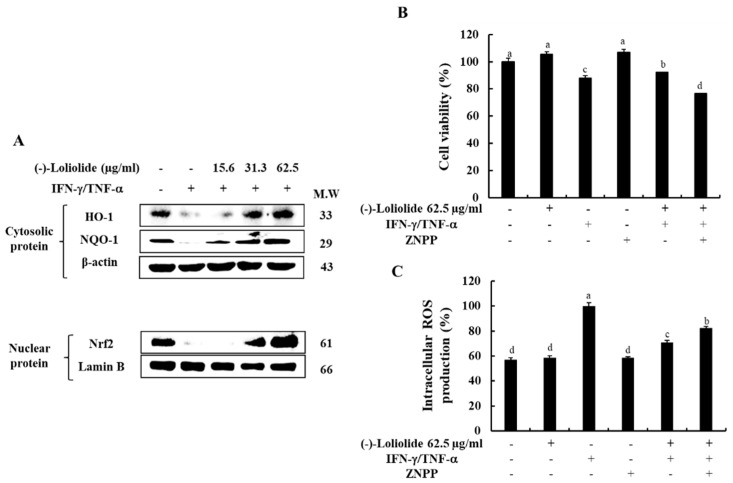
Effect of (–)-loliolide on the activation of the Nrf2/HO-1/NQO-1 signaling pathway (**A**) and influence of HO-1 inhibition on cell viability (**B**) and intracellular ROS production (**C**) in IFN-γ/TNF-α-stimulated HaCaT keratinocytes. The results represent data from three independent experiments (n = 3), and the values are indicated as the mean ± SE. Error bars with different letters are significantly different (*p* < 0.05).

We investigated the effects of (–)-loliolide on the Nrf2/HO-1 signaling, known as an antioxidant system in IFN-γ/TNF-α in HaCaT keratinocytes. Figure 5A shows that IFN-γ/TNF-α stimulation down-regulated the expression level of HO-1 in the cytosol, as well as the expression level of Nrf2 in the nucleus, whereas they were markedly increased by the pretreatment of (–)-loliolide. Additionally, we evaluated whether the HO-1 activation is associated with the cytoprotective effects of (–)-loliolide in IFN-γ/TNF-α-stimulated HaCaT keratinocytes. The results showed that (–)-loliolide has the cytoprotective effect of markedly improving the cell viability by reducing the intracellular ROS production in IFN-γ/TNF-α-stimulated HaCaT keratinocytes. Interestingly, they are significantly abolished by the pretreatment of ZnPP, a HO-1 inhibitor (Figure 5B,C). These results suggest that the activation of HO-1 is required for the cytoprotective effects of (–)-loliolide in IFN-γ/TNF-α-stimulated HaCaT keratinocytes.

## 4. Discussion

Marine algae are rich in bioactive compounds such as phlorotannins, fucoidans, and phycocolloids, and hence they can be used as pharmaceutical foods as well as functional foods [21]. In particular, *S. horneri* extract and its active compounds including (–)-loliolide, fucosterol, and sargachromenol have demonstrated various physiological activities such as anti-allergic, antioxidant, and anti-inflammatory activities [22,23]. Especially, our previous studies have mentioned (–)-loliolide leads to the anti-inflammatory effects in LPS-stimulated macrophages and fine dust-activated fibroblast [24]. Despite the discovery of its various beneficial activity, there is no report about effect (–)-loliolide on the inflammation response in the activated keratinocytes known as the first line of defense mechanism in the skin.

Thus, in the present study, we examined the effects of (–)-loliolide derived from *S. horneri* on the IFN-γ/TNF-α stimulation-induced inflammatory responses in HaCaT keratinocytes.

First, we evaluated the cytotoxicity to confirm the safety of (–)-loliolide purified from *S. horneri*. As indicated in Figure 1, (–)-loliolide significantly increased the cell viability by decreasing the intracellular ROS production in IFN-γ/TNF-α-stimulated HaCaT keratinocytes.

Normally, IL-25, IL-33, and TSLP play important roles as triggers that promote the proliferation of Th2 cells and their cytokine production [25] in the epidermis of the skin. In particular, TSLP directly activates naïve T-lymphocytes and induces the production of IL-1β, IL-4, IL-5, IL-13, and TNF-α [26]. Inflammatory cytokines, such as IL-4, IL-6, IL-13, IFN-γ, and TNF-α are known to mediate inflammatory or allergic reactions upon secretion from skin keratinocytes when exposed to external stimulation [14]. Previous studies have also showed that when HaCaT keratinocytes were stimulated with UVB or IFN-γ/TNF-α, the levels of inflammatory cytokines, such as IL-4, IL-6, IL-13, IFN-γ, and TNF-α were significantly increased; a phenomenon that was consistent with our results [19,27]. The representative inflammatory chemokines including Eotaxin, MDC, RANTES and TARC, regulate the recruitment of leukocytes to the site of inflammation and are involved in the onset of inflammation [28]. In the current study, we also found that (–)-loliolide effectively reduced the expression and secretion of inflammatory cytokines (IL-1β and IL-5) and chemokines (Eotaxin, MDC, RANTES, and TARC) by decreasing the transcript-level expression of epidermal-derived initial cytokines (IL-25, IL-33, and TSLP) in IFN-γ/TNF-α-stimulated HaCaT keratinocytes. With these results, we suggest that (–)-loliolide inhibited the inflammation responses in the activated skin keratinocytes (Figure 2).

The activation and production of inflammatory cytokines and chemokines are involved in triggering multiple signaling pathways including NF-κB and MAPK signaling [29]. A previous study has reported that the p38 MAPK pathway plays an important role in the UVB-induced inflammatory response in mice with hairless skin [30]. In addition, phosphorylation of ERK subsequently triggers the expression of many transcription factors involved in inflammation [31]. Our study also proved that (–)-loliolide inhibits the phosphorylation of p38 MAPK and ERK in IFN-γ/TNF-α-stimulated HaCaT keratinocytes (Figure 4A). NF-κB, a downstream molecule of MAPK is known to regulate the expression of genes, enzymes, and adhesion molecules involved in chronic inflammatory diseases [32]. Under normal conditions, NF-κB, which consists of p65 and IκBα complexes is localized in the cytosol. However, the phosphorylation and release of IκBα from the NF-κB complex results in the activation and translocation of p65 into the nucleus. After activation, NF-κB led to the activation of inflammation-related factors, such as cytokines and chemokines. In this study, IFN-γ/TNF-α stimulation induced the phosphorylation of p65 and IκBα and translocation of p65, whereas (–)-loliolide effectively inhibited these events (Figure 4B). These results indicated that (–)-loliolide markedly suppressed the expression and/or secretion of inflammatory cytokines and chemokines by inactivating the MAPK and NF-κB signaling pathways in IFN-γ/TNF-α-stimulated HaCaT keratinocytes.

Under conditions of oxidative stress and inflammation, HO-1 acts as an important modulator in cell protection [33]. HO-1 is an antioxidant-related protein known to effectively downregulate the expression of molecules related to the development of oxidative stress (e.g., ROS and nitric oxide (NO)) [34]. In addition, activation of the Nrf2/HO-1 signaling pathway has been demonstrated to be effective at suppressing inflammation [35]. Previous studies have demonstrated that activation of the Nrf2 pathway decreased the productions of ROS and NO and suppressed in inflammatory cytokines, such as IL-4 and IL-6 by activating of NF-κB signaling in LPS-stimulated macrophages [14,33]. In this study, we discovered the IFN-γ/TNF-α stimulation inhibited the Nrf2/HO-1 signaling in HaCaT keratinocytes as decreased the expression levels of HO-1 and NQO-1 in the cytosol as well as the expression level of Nrf2 in the nucleus. Interestingly, the pretreatment of (–)-loliolide activated the Nrf2/HO-1 signaling in IFN-γ/TNF-α-stimulated HaCaT keratinocytes by increasing the expression levels of HO-1, NQO-1 and Nrf2 (Figure 5). Next, we checked whether the capacities of (–)-loliolide on the activation of HO-1 signaling is associated to its anti-inflammatory effect in IFN-γ/TNF-α-stimulated HaCaT keratinocytes. Indeed, the inhibition of HO-1 signaling following the application of ZnPP (a HO-1 inhibitor) abolished the cell viability, and the intracellular ROS production improved by treatment with (–)-loliolide in IFN-γ/TNF-α-stimulated HaCaT keratinocytes (Figure 5B,C). With these results, we demonstrate that the activation of HO-1 signaling is required for the anti-inflammatory effects of (–)-loliolide via the inhibition of oxidative stress and inflammation caused by the stimulation of IFN-γ/TNF-α in HaCaT keratinocytes.

## 5. Conclusions

Taken together, these results revealed that (–)-loliolide effectively suppressed the oxidative stress and the inflammation by activating Nrf2/HO-1 signaling in IFN-γ/TNF-α-stimulated HaCaT keratinocytes. In addition, this study suggests that (–)-loliolide may potentially be used as a component in cosmeceuticals and functional foods to attenuate oxidative stress and inflammatory responses.

## Data Availability

Data is contained within the article.
